# Involvement of BDNF/TrkB and ERK/CREB axes in nitroglycerin-induced rat migraine and effects of estrogen on these signals in the migraine

**DOI:** 10.1242/bio.021022

**Published:** 2016-11-14

**Authors:** Jiu-Qing Guo, Hui-Hui Deng, Xiao Bo, Xiao-Su Yang

**Affiliations:** Department of Neurology, Xiangya Hospital, Central South University, 87 Xiangya Road, Changsha, Hunan 410008, People's Republic of China

**Keywords:** BDNF, TrkB, ERK, CREB, Migraine, Estrogen

## Abstract

Migraine is a highly prevalent headache disorder, especially in women. Brain-derived neurotrophic factor (BDNF) and its receptor tropomyosin receptor kinases (TrkB), as well as extracellular signal-regulated kinase (ERK) and its downstream target c-AMP-responsive element binding protein (CREB) are strongly associated with the transmission of nociceptive information. However, the involvement of these substances in migraine has rarely been examined. In the present study, intraperitoneal injection of nitroglycerin (NTC) successfully induced rat migraine attack, as evidenced by behavioral testing. The location and abundance of these substances in the migraine model were determined by immunohistochemistry, real-time polymerase chain reaction (RT-PCR), western blot and enzyme-linked immunosorbant assays (ELISA). Results showed that BDNF, TrkB, phosphor(p)-ERK and p-CREB were up-regulated in the brain neurons of both male and female rats with NTG-induced migraine compared to non-migraine control, whereas their expression levels were decreased in headache-free intervals of the migraine compared to migraine attacks. Estrogen is an important contributor to migraine. Female ovariectomized rats showed significant reduction in the expression of BDNF, TrkB, p-CREB and p-ERK in both attacks and intervals of NTG-induced migraine, relative to rats that have their ovaries. But, intraperitoneal administration of exogenous estrogen recovered their expression in ovariectomized rats. Collectively, this study unveiled a positive correlation of BDNF/TrkB and ERK/CREB axes in NTG-induced migraine and promoting effects of estrogen on their signals in the migraine. These findings contribute to further understanding the pathogenesis of migraine in the molecular basis.

## Abstract (precis)

**Summary:** This study unveiled a positive correlation of BDNF/TrkB and CREB/ERK axes in NTG-induced migraine and the promoting effects of estrogen on their signals in the migraine.

## INTRODUCTION

Migraine is a common brain disorder, adversely affecting about 11% of global population (Fischer et al., 2012). It is characterized by predominantly unilateral pulsating head pain with a series of complications, including nausea, vomiting, hypersensitivity to light, cognitive, emotional and motor disturbances, as well as sound, smell and visual disorders (Gölöncsér and Sperlágh, 2014). The pathological mechanisms of migraine are very complicated, which have been associated with neurogenic inflammation, central sensitization, cortical spreading depression, oxidative stress and trigeminovascular damage (Fischer et al., 2012; Borkum, 2016).

In addition to these mechanisms, several lines of evidence highlight that estrogen is probably an important contributor to migraine. Human migraine has been found to be two to three times more prevalent in females than in males (Scharfman and MacLusky, 2008). The incidence of migraine in women is higher in the reproductive age with a peak serum estrogen level, but lower in the time prior to puberty and after menopause (estrogen bottoms at these two periods), as compared to other age phases (Scharfman and MacLusky, 2008). Reports from more than 50% of female patients show that migraine attacks are positively correlated with their menstrual cycle (Merki-Feld et al., 2015). These data imply a positive association between migraine attacks and fluctuations of serum estrogen level. Supporting this concept, epidemiological studies manifest that combined hormonal contraceptives that mainly contain estrogen initiate or worsen migraine and headache in predisposed women (Merki-Feld et al., 2015). Ongoing research finds that ovariectomy in female rats attenuates neuronal activation in nucleus trigeminalis caudalis of the brainstem as well as in paraventricular nucleus and supraoptic nucleus of the hypothalamus, whereas chronic intraperitoneal administration of estrogen restores the activation to the neuron (Greco et al., 2013). Activation of central trigeminal neurons within the trigeminal nucleus caudalis is crucial for the development of throbbing in the initial phase of migraine (Greco et al., 2013). Although estrogen has been proposed to play a critical role in the pathogenesis of migraine, the underlying molecular basis is not well understood.

Brain-derived neurotrophic factor (BDNF) is regarded as a neurotrophin within the central and the peripheral nervous system, controlling neuronal development and differentiation (Fischer et al., 2012). Recent findings reveal that BDNF also functions as a positive modulator in pain signaling. It has been found that BDNF regulates plasticity of synapses in trigeminal nociceptive pathways partly through affecting the efficiency of glutamatergic and GABAergic/glycinergic synapses (Buldyrev et al., 2006). BDNF has been shown to participate in the pathogenesis of migraine comorbidities, such as epilepsy and depression (Yang et al., 2016), and a strong relationship between migraine and depression in pathogenetic mechanisms has been confirmed by previous documents (Yang et al., 2016). Thus, BDNF may also be involved in the generation and/or modulation of migraine. Biological activities of BDNF rely on the binding to tropomyosin receptor kinases (TrkB) that couples to an array of signal transduction (Scharfman and MacLusky, 2008). BDNF has been reported to be regulated by estrogen. An estrogen-sensitive response element has been identified on the *BDNF* gene (Scharfman and MacLusky, 2008). This report leads to the conjecture that BDNF/TrkB signaling mediates estrogen actions in migraine.

The c-AMP-responsive element binding protein (CREB) is a transcription factor that plays a critical role in adaptive neuronal responses, in addition to the complex functions in regulation of learning and memory. CREB is phosphorylated at serine 133 (p-CREB) and activated, upon stimulation of pain-producing signals, like those mediated by extracellular signal-regulated kinase (ERK) (Bhatt et al., 2015). Activated CREB can further induce activation of c-Fos that is a marker of neuronal activation within brainstem and spinal nociceptive pathways (Bhatt et al., 2015). Thus, CREB very likely serves as an important mediator in the transmission of nociceptive information. BDNF is also a downstream target of CREB according to the finding that CREB positively controls gene expression of BDNF via binding to the gene promotor region (Tong et al., 2001; Zha et al., 2001; Hong et al., 2008). Interestingly, there exists evidence indicating that BDNF mediates CREB phosphorylation because using anti-BDNF antibody to block actions of BDNF effectively prevents CREB phosphorylation (Simonetti et al., 2008). These data suggest that there is a dual directional regulation mechanism between BDNF and CREB.

This study initially investigated the involvement of BDNF, TrkB, p-ERK and p-CREB in the migraine attacks that were induced by nitroglycerin (NTG) injection in rats. Systemic administration of NTG, a nitric oxide donor, has been established to trigger a migraine-like headache in both healthy subjects and patients suffering from primary headaches (Gölöncsér and Sperlágh, 2014; Greco et al., 2014). NTG infusion in rats can also give rise to a migraine-like response, thus it is a universally accepted model that mimics human migraine. Considering that estrogen has promoting effects on migraine, this study subsequently investigated the influence of estrogen on expression of BDNF, TrkB, p-CREB and p-ERK in the nitroglycerin (NTG)-induced migraine rat model to determine whether these factors mediate estrogen actions in migraine. The aim of this study was to further reveal the pathogenesis of migraine in the molecular basis.

## RESULTS

### NTG injection induced recurrent migraine in both male and female rats

NTG infusion is amongst the most widely used and accepted approaches to induce migraine attacks in both animals and human. 4 h after the fifth injection with NTG, rats presented typical manifestations of migraine, like painful facial action, scratching head and cages, red ear, tail flick, and photophobia, suggesting that NTG injection (i.p.) effectively led to migraine attacks. All the symptoms were basically subsided on the second day, which suggested rats were in the headache-free interval. We recorded the start and end times when rats showed red ear after administration of NTG and calculated the duration for statistical analysis (Table 1). The male rats with a typical manifestation of red ear were observed ∼24 min after treatment with NTG. There was a little delay for the manifestation of red ear in the female rats. The starting time was ∼28 min after NTG treatment. This manifestation lasted for ∼275 and ∼291 min, respectively, in the male and female rats (both P<0.01 vs control). No significant difference was observed between the male and female rat for the duration of red ear. The number of times rats scratched their head and cages after NTG treatment were noted in Tables 2 and 3, respectively.

**Table 1.**
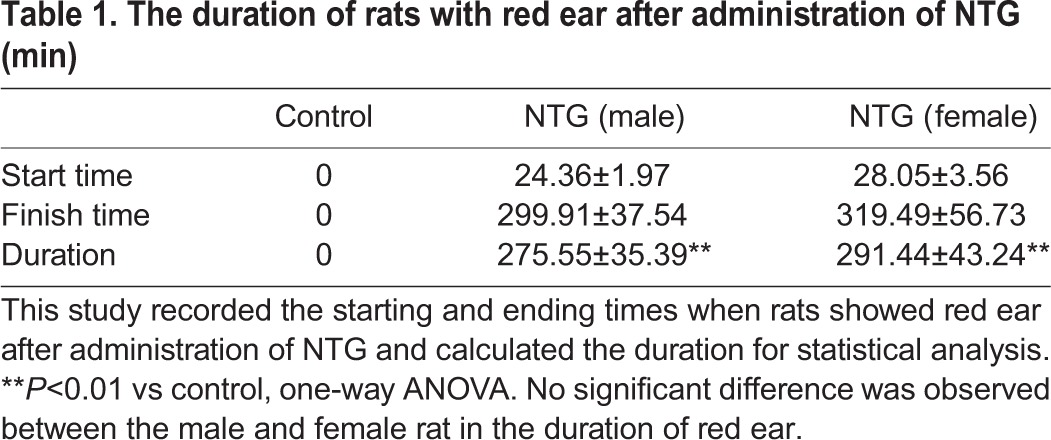
**The duration of rats with red ear after administration of NTG (min)**

**Table 2.**
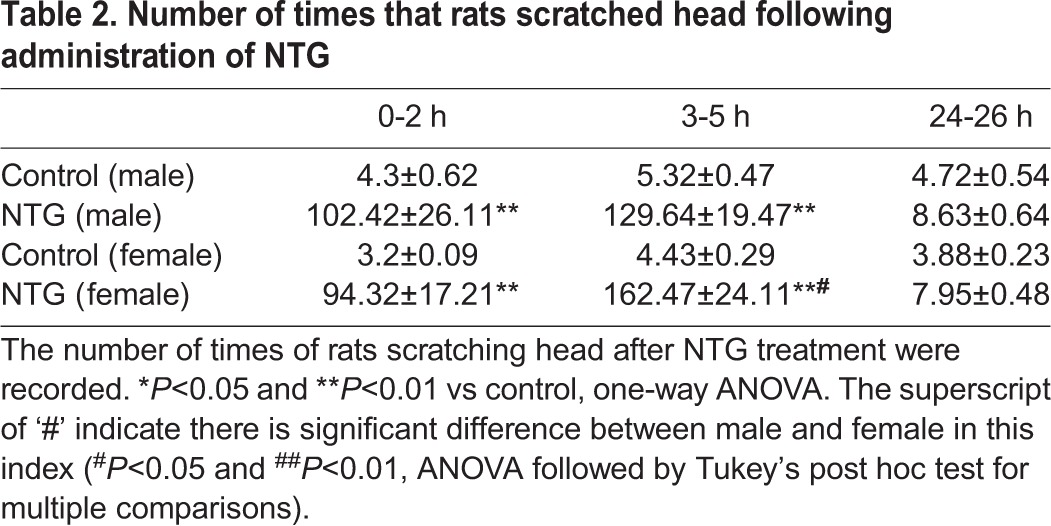
**Number of times that rats scratched head following administration of NTG**

**Table 3.**
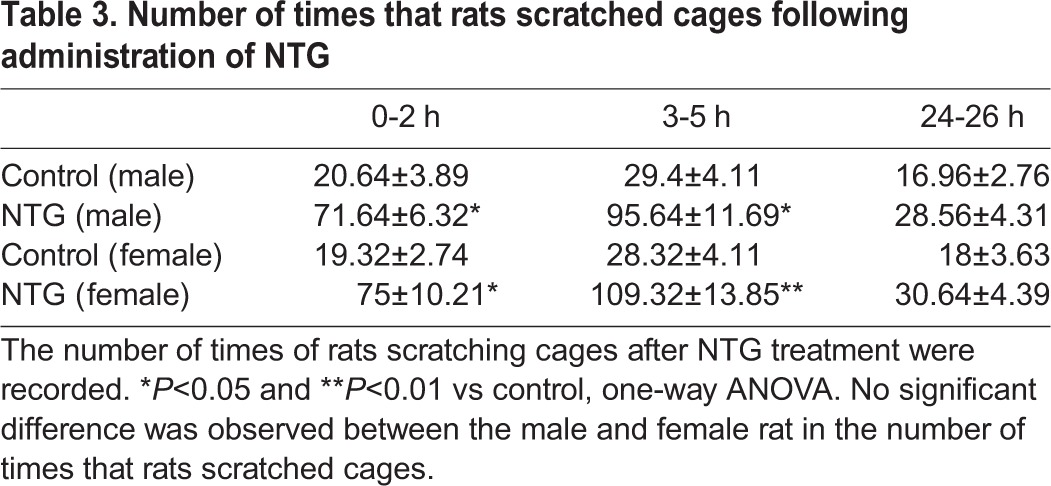
**Number of times that rats scratched cages following administration of NTG**

There was notable increase in the number of times that rats (both male and female) scratched their head in the ∼0-2 h (P<0.01 vs control) and ∼3-5 h (P<0.01 vs control), but not in the ∼24-26 h, after NTG administration. We noticed that female rats scratched their head more times than male rats during ∼3-5 h after the NTG treatment (P<0.05). The number of times that the rats scratched the cages also had dramatic increase in ∼0-2 h (P<0.05) and ∼3-5 h (P<0.05 in male rats and P<0.01 in female rats) after NTG administration, compared to those of control rats in similar time frames.

### Expression of BDNF, TrkB, ERK and CREB in the migraine attacks and headache-free intervals

**Fig. 1.**
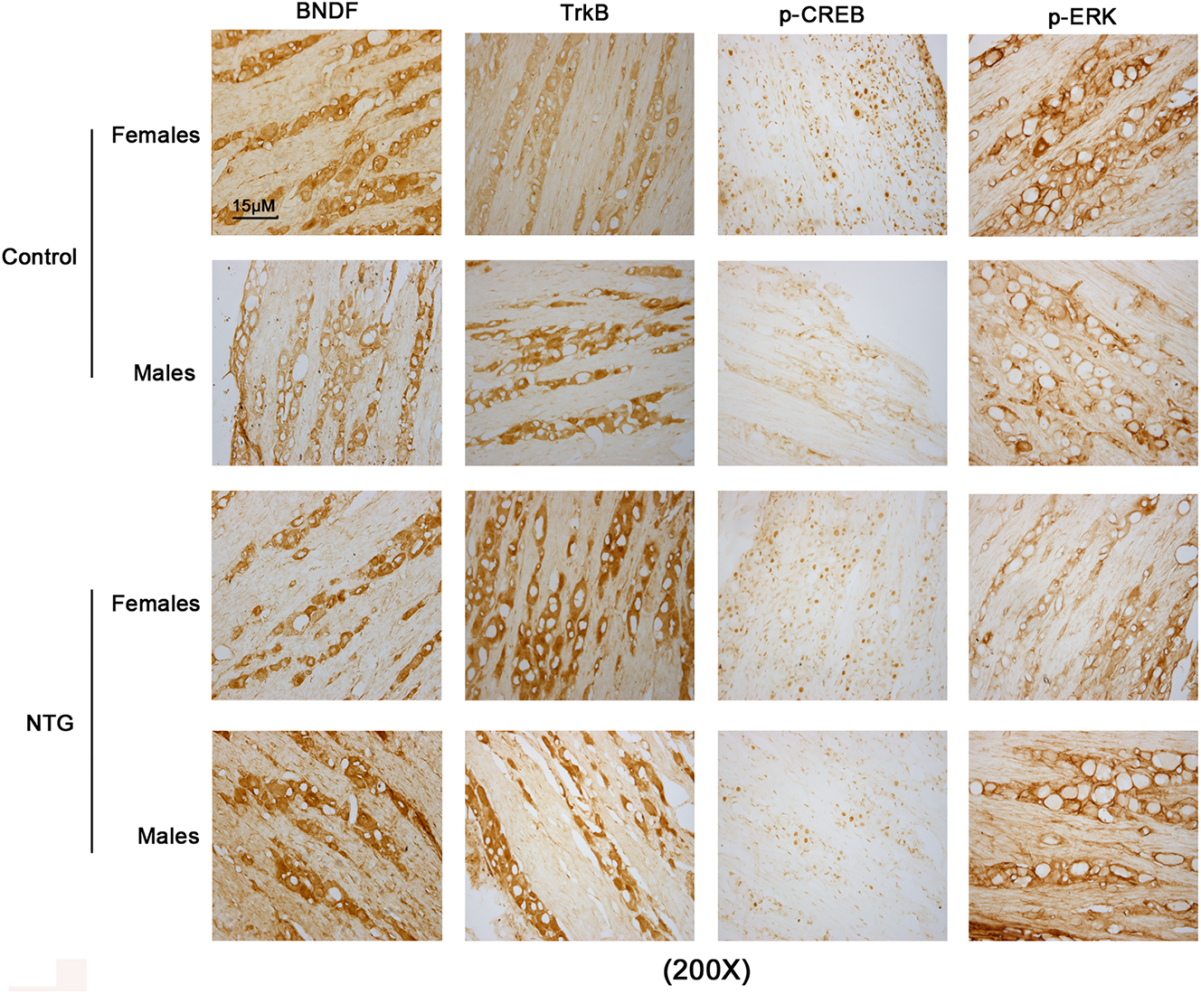
**IHC detection mapped expression of BDNF, TrkB, p-ERK and p-CREB in the brain of rats that were subjected to injection with NTG or vehicle.** 24 male and 24 female Sprague–Dawley rats received intraperitoneal (i.p.) injection of the 10 mg/kg NTG or its vehicle once a week. The rats were sacrificed during the migraine attacks (4 h after the fifth injection) or the headache-free intervals (24 h after the fifth injection). After rats were anaesthetized with ketamine and xylazine (i.p.), the whole brain was removed and subjected to IHC detection. BDNF, brain-derived neurotrophic factor; TrkB, tropomyosin receptor kinases; p-ERK, phosphorylated extracellular signal-regulated kinase; p-CREB, phosphorylated c-AMP-responsive element binding protein.

To understand the function of BDNF, TrkB, ERK and CREB in migraine, we initially mapped their expression in the brain of rats that were subjected to injection with NTG or vehicle. Immunohistochemistry (IHC) has high sensitivity to detect and locate targeted proteins in tissues. IHC detection showed that BDNF and TrkB were mainly expressed in neurons, no matter if rats were injected with NTG or vehicle (Fig. 1). Phosphorylation of serine-133 is a critical event in CREB activation. p-CREB was mainly present in the nucleus of neurons, and the location of p-CREB seemed to be unaffected by the NTG injection. p-ERK distributed in the whole brain cells, especially in neurons. Treatment with NTG had no effect on the location of p-ERK in the brain. No obvious difference was seen in the position of these proteins between female and male rats.

**Fig. 2.**
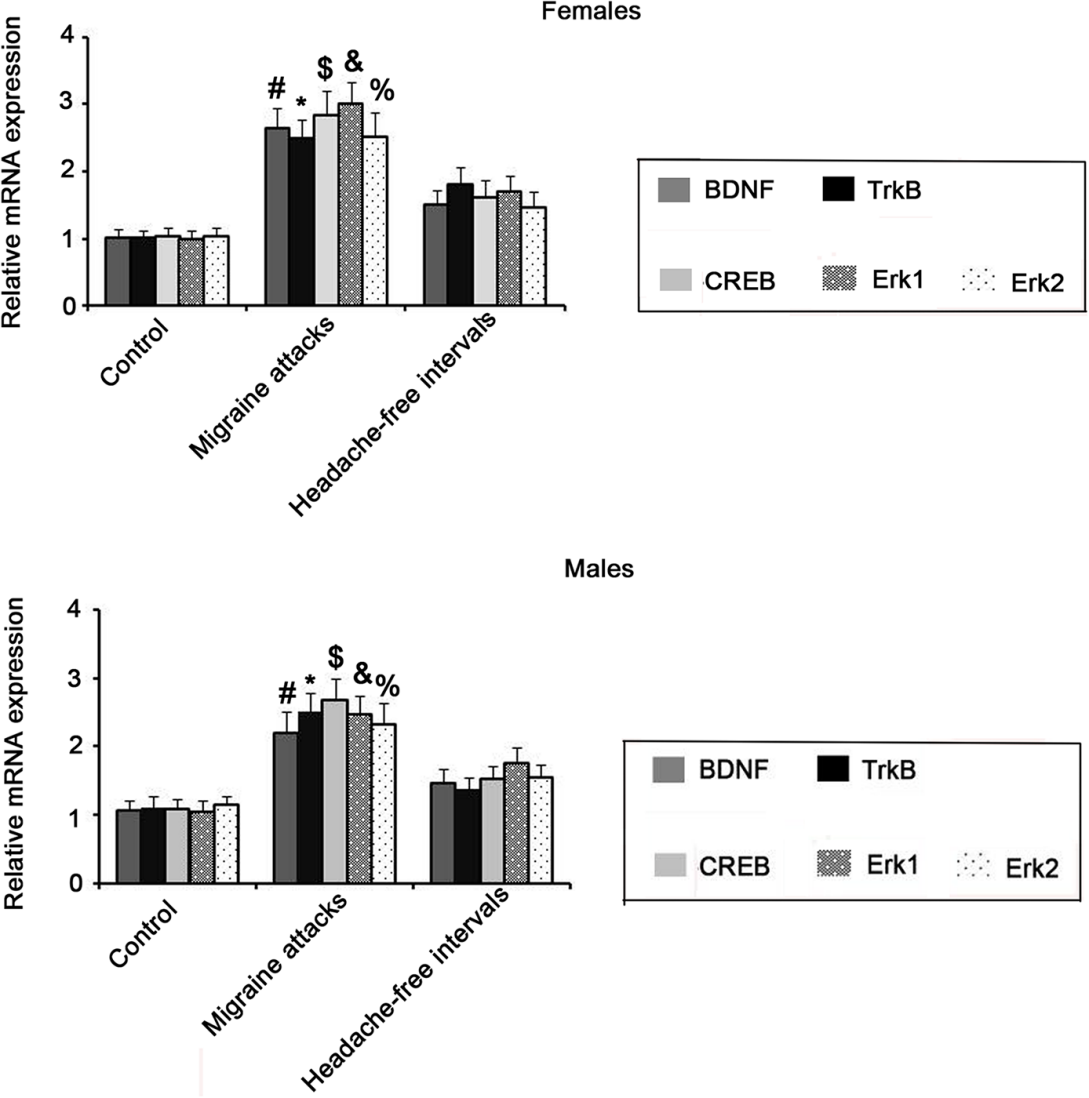
**RT-PCR assay showed mRNA expression levels of BDNF, TrkB, CREB and ERK in the brain of rats that were injected with NTG or vehicle.** 24 male and 24 female Sprague–Dawley rats received intraperitoneal (i.p.) injection of the 10 mg/kg NTG or its vehicle once a week. The rats were sacrificed during the migraine attacks (4 h after the fifth injection) or the headache-free intervals (24 h after the fifth injection). After rats were anaesthetized with ketamine and xylazine (i.p.), the whole brain was removed and subjected to RT-PCR assay. Bars with the marks #, $, %, * and & indicate significant difference between treatment groups and control groups (*P*<0.05, *n*=6, one-way ANOVA). BDNF, brain-derived neurotrophic factor; TrkB, tropomyosin receptor kinases; ERK, extracellular signal-regulated kinase; CREB, c-AMP-responsive element binding protein.

**Fig. 3.**
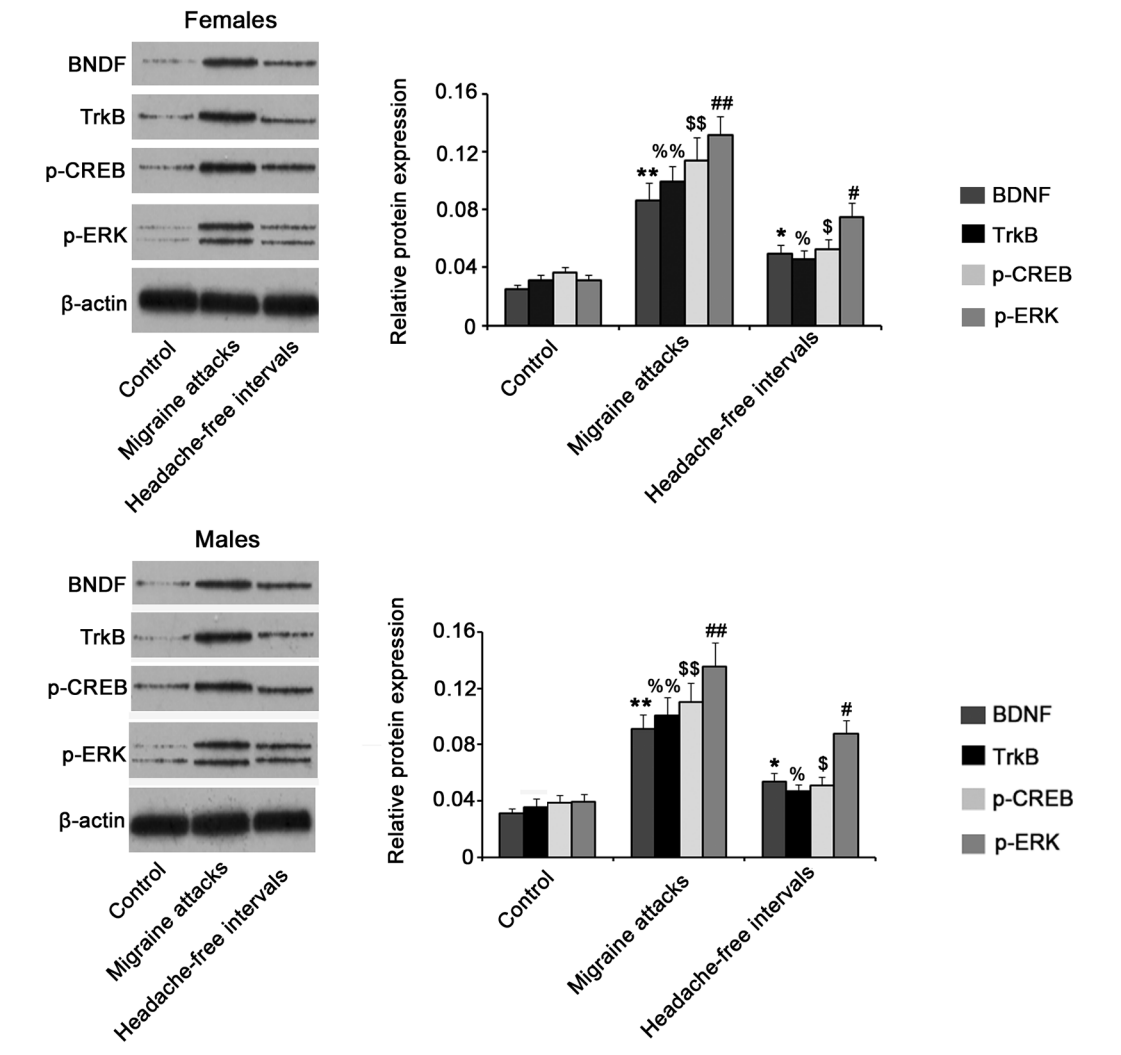
**Western blot assay showed protein expression levels of BDNF, TrkB, p-CREB and p-ERK in the brain of rats that were injected with NTG or vehicle.** 24 male and 24 female Sprague–Dawley rats received intraperitoneal (i.p.) injection of the 10 mg/kg NTG or its vehicle once a week. The rats were sacrificed during the migraine attacks (4 h after the fifth injection) or the headache-free intervals (24 h after the fifth injection). After rats were anaesthetized with ketamine and xylazine (i.p.), the whole brain was removed and subjected to western blot assay. Bars with the marks #, $, %, * and & indicated significant difference between treatment groups and control groups (*P*<0.05, *n*=6). Bars with the duplicated marks indicated *P*<0.01. BDNF, brain-derived neurotrophic factor; TrkB, tropomyosin receptor kinases; p-ERK, phosphorylated extracellular signal-regulated kinase; p-CREB, phosphorylated c-AMP-responsive element binding protein.

RT-PCR and western blot assays provide suitable methods to quantify mRNA and protein expression levels of target genes, respectively, in tissues. With RT-PCR assay, mRNA expression of BDNF, TrkB, CREB and ERK (including the ERK1 and ERK2 subunits) were found to be significantly up-regulated in the brain of both female and male rats with migraine attack compared to non-migraine control rats (*P*<0.05, Fig. 2). There was no significant difference between female and male rats in the expression of these proteins during migraine attack. Although mRNA expression of BDNF, TrkB, CREB, ERK1 and ERK2 were higher in the headache-free intervals of migraine than in non-migraine control, there was no statistical difference. Data from western blotting showed that NTG-induced migraine resulted in dramatic increase in BDNF, TrkB, p-CREB and p-ERK expression, no matter in the attack (*P*<0.01) and interval of migraines (*P*<0.05), compared to control group (Fig. 3). Statistical analysis did not show any difference between female and male rats in protein expression of these proteins.

### BDNF was increased in the serum of rates following the NTG administration

**Fig. 4.**
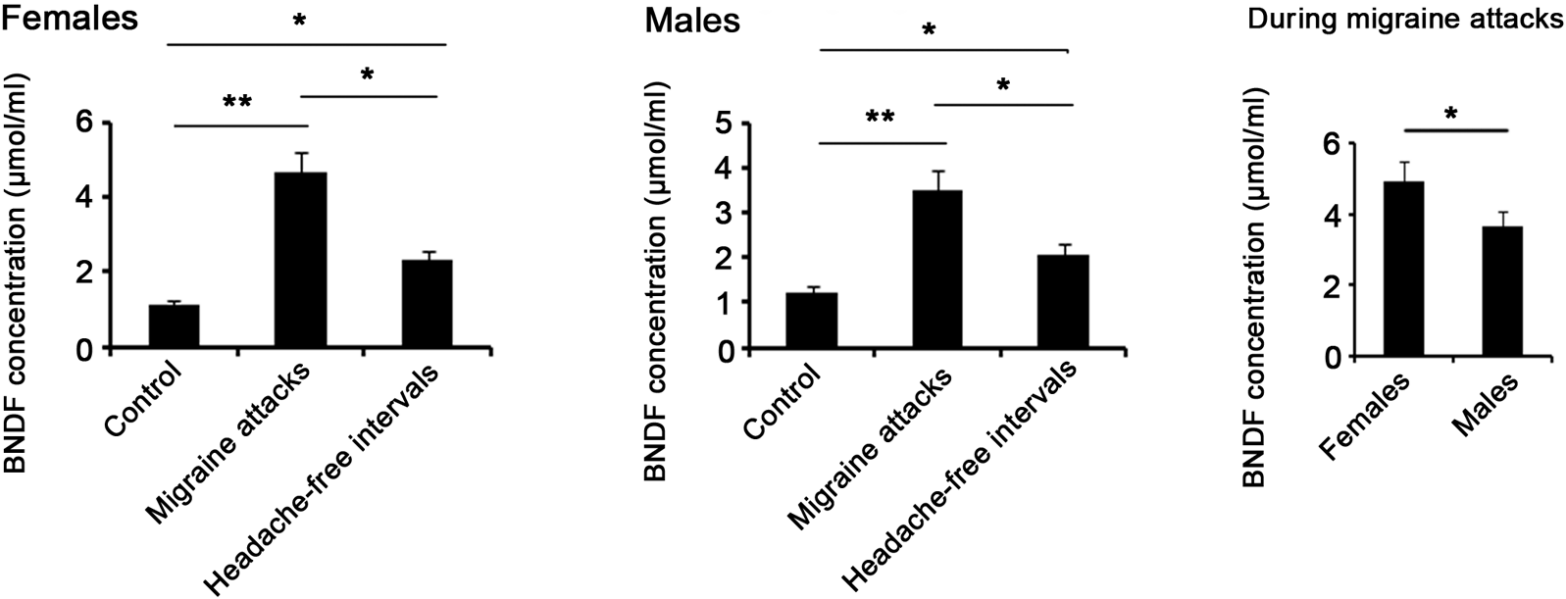
**Serum BDNF level in rats that were injected with NTG or vehicle.** 24 male and 24 female Sprague–Dawley rats received intraperitoneal (i.p.) injection of the 10 mg/kg NTG or its vehicle once a week. The rats were sacrificed during the migraine attacks (4 h after the fifth injection) or the headache-free intervals (24 h after the fifth injection). Rats' blood was drawn from the vena cava before the sacrifice, and then centrifuged at 3000 ***g*** for 10 min at 4°C to separate serum. The level of BDNF in serum was quantified by ELISA. *P<0.05, **P<0.01 between each group (*n*=12, ANOVA followed by a Tukey's post hoc test for multiple comparisons). BDNF, brain-derived neurotrophic factor.

A previous study noted that BDNF was released in cerebrospinal fluid and blood after inducing by trigeminal stimulation and nociceptive inputs (Fischer et al., 2012). Using the ELISA method, we observed remarkable increase in serum BDNF level in migraine attack (*P*<0.01; Fig. 4), compared to non-migraine control. Serum BDNF showed higher levels in female than male rats in migraine attack (*P*<0.05). Although BDNF had significant reduction in headache-free intervals of migraine relative to migraine attack (*P*<0.05), BDNF in headache-free intervals was higher than that in non-migraine control group (*P*<0.05).

### Effect of estrogen on expressions of BDNF, TrkB, ERK and CREB in NTG-induced migraine

**Fig. 5.**
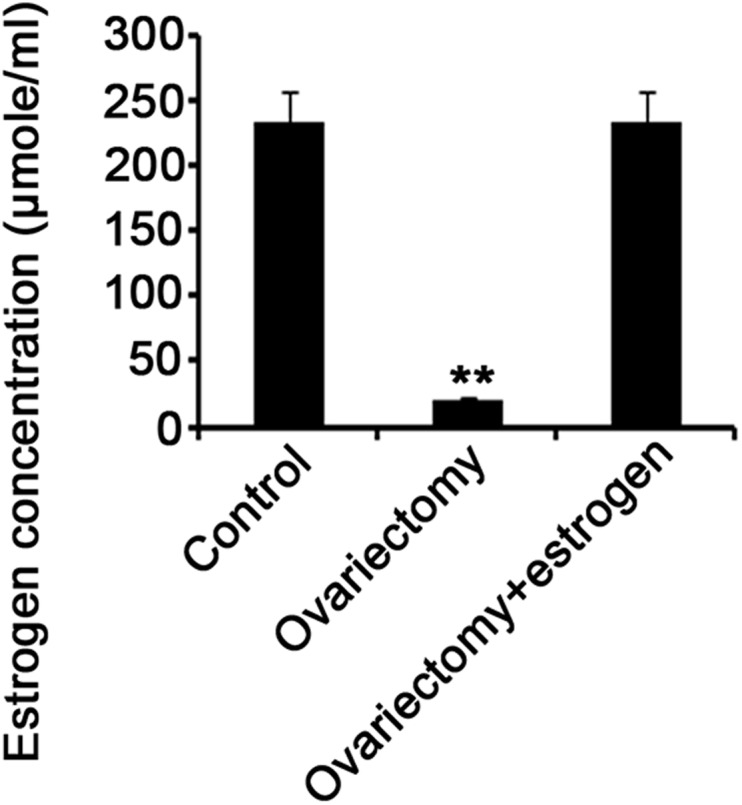
**Serum estrogen concentration in rats following different treatments.** 24 female Sprague–Dawley rats with ovariectomy underwent intraperitoneal administration of estrogen or its vehicle. 12 rats of sham-operated group only underwent the resection of a bit of fat around bilateral ovaries. All these rats were injected with 10 mg/kg NTG once a week and the injection was repeated five times. Rats' blood was drawn from the vena cava before the sacrifice, and then centrifuged at 3000 ***g*** for 10 min at 4°C to separate serum. Serum estrogen concentration was quantified by ELISA. **P<0.01 between each group (*n*=12, one-way ANOVA).

**Fig. 6.**
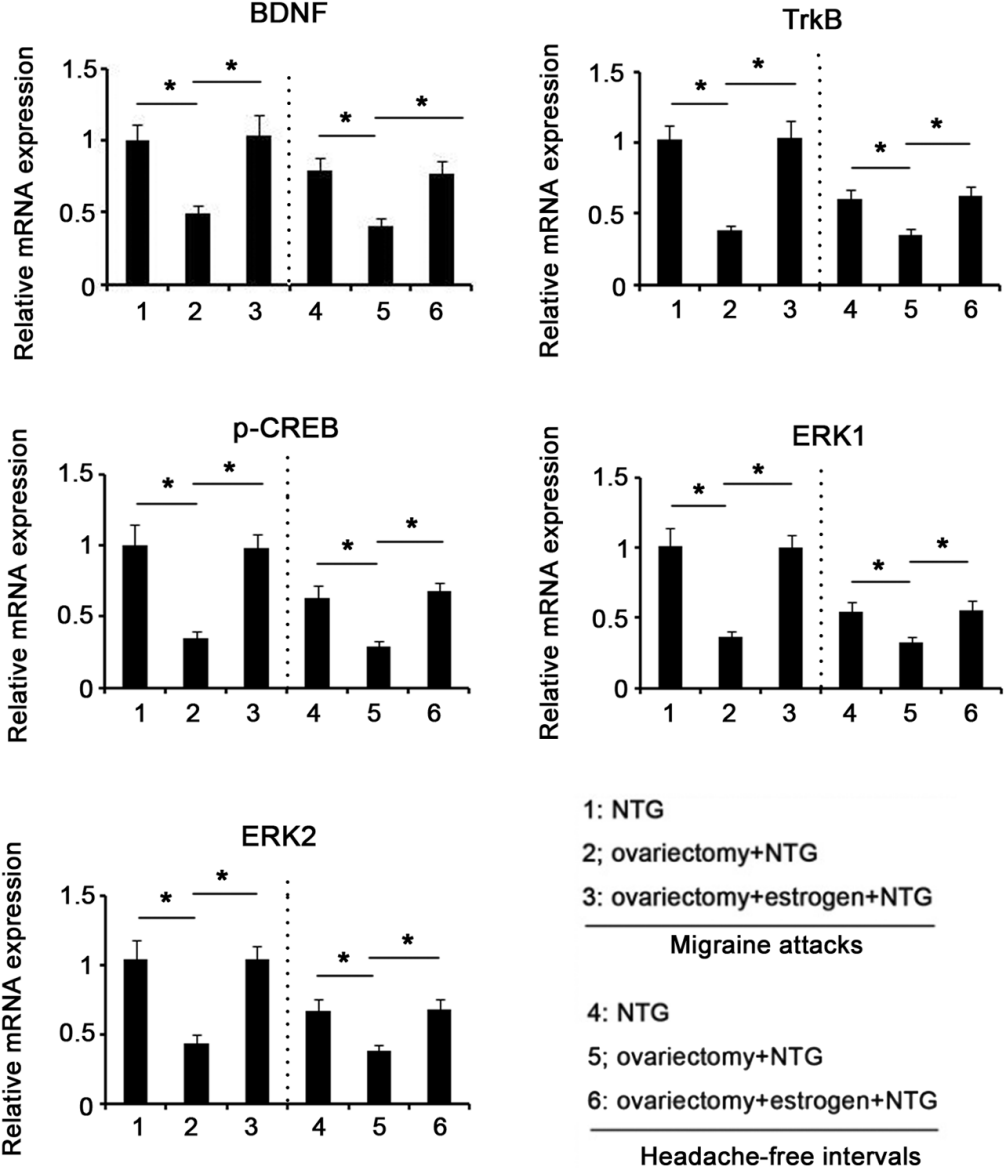
**mRNA expression levels of BDNF, TrkB, CREB and ERK in the brain of rats after different treatments.** 24 female Sprague–Dawley rats with ovariectomy underwent intraperitoneal administration of estrogen or its vehicle. 12 rats of sham-operated group only underwent the resection of a bit of fat around bilateral ovaries. All these rats were injected with 10 mg/kg NTG once a week and the injection was repeated five times. The rats were sacrificed during the migraine attacks (4 h after the fifth injection) or the headache-free intervals (24 h after the fifth injection). After rats were anaesthetized with ketamine and xylazine (i.p.), the whole brain was removed and subjected to RT-PCR assay. *P<0.05 between each group (*n*=12, ANOVA followed by a Tukey's post hoc test for multiple comparisons). BDNF, brain-derived neurotrophic factor; TrkB, tropomyosin receptor kinases; ERK, extracellular signal-regulated kinase; CREB, c-AMP-responsive element binding protein.

**Fig. 7.**
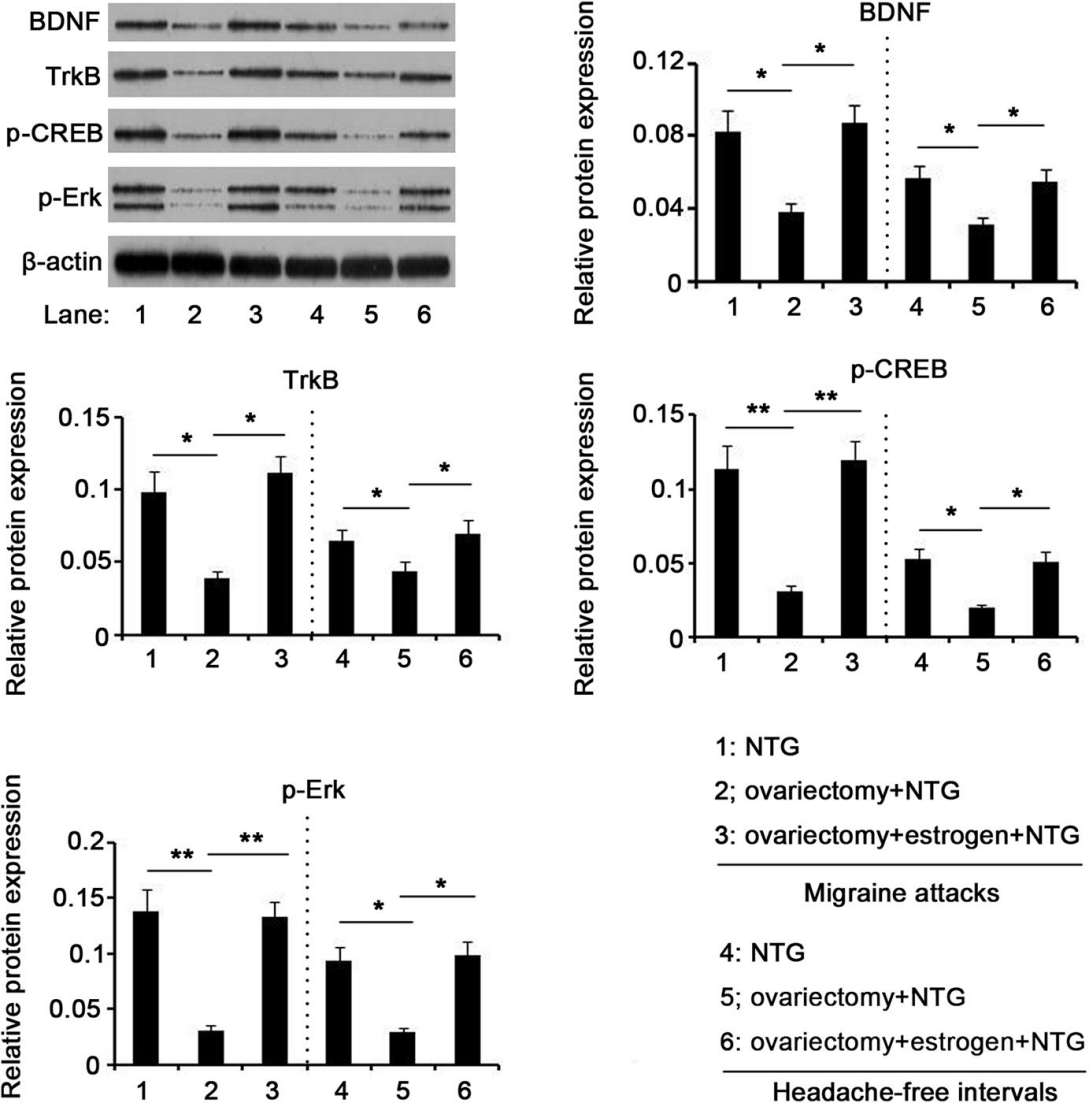
**Protein expression levels of BDNF, TrkB, p-CREB and p-ERK in the brain of rats after different treatments.** 24 female Sprague–Dawley rats with ovariectomy underwent intraperitoneal administration of estrogen or its vehicle. 12 rats of sham-operated group only underwent the resection of a bit of fat around bilateral ovaries. All these rats were injected with 10 mg/kg NTG once a week and the injection was repeated five times. The rats were sacrificed during the migraine attacks (4 h after the fifth injection) or the headache-free intervals (24 h after the fifth injection). After rats were anaesthetized with ketamine and xylazine (i.p.), the whole brain was removed and subjected to RT-PCR assay. *P<0.05, **P<0.01 between each group (*n*=12, ANOVA followed by a Tukey's post hoc test for multiple comparisons). BDNF, brain-derived neurotrophic factor; TrkB, tropomyosin receptor kinases; p-ERK, phosphorylated extracellular signal-regulated kinase; p-CREB, phosphorylated c-AMP-responsive element binding protein.

Estrogen is an important contributor to migraine, but the underlying mechanisms are not well understood. To understand whether estrogen had an effect on expression of BDNF, TrkB, p-ERK and p-CREB in NTG-induced migraine, we established an ovariectomized rat model prior to injection with NTG. Ovariectomy resulted in dramatic reduction in serum estrogen level, as observed by ELISA test (*P*<0.01, Fig. 5); but exogenous estrogen administration recovered serum estrogen level. PCR detection showed that ovariectomy led to a remarkable decrease in mRNA expression of BDNF, TrkB, CREB, ERK1 and ERK2 in both attacks and intervals of migraines induced by NTG infusion (*P*<0.05, Fig. 6), whereas restoration of serum estrogen level via estrogen administration reversed their mRNA expression. Compared to rats that had intact ovaries, ovariectomized rats showed decreased protein expression of BDNF, TrkB, p-CREB and p-ERK in both attacks and intervals of migraines (*P*<0.05, Fig. 7), while the decreased protein expression was recovered after exogenous estrogen administration.

## DISCUSSION

Migraine is among the most common primary headache disorders contributing to significant disability and reduced quality of life (Fischer et al., 2012), and despite its prevalence and impact, the treatment options continue to be limited. BDNF serves as an important modulator of central and peripheral pain responses, but the role it has in migraine and its pathophysiology is incompletely understood. In the present study, BDNF and its receptor TrkB were up-regulated in the brain neurons of both male and female rats with NTG-induced migraine compared to non-migraine control, as evidenced by PCR and western blot assays, while their expression had a notable decrease in headache-free intervals of migraine compared to the migraine episodes. Inconsistently, Cho et al. found that the expression of BDNF and TrkB in the hippocampus were decreased immediately after isoflurane-induced transient anterograde amnesia, but were increased 2 h later (Cho et al., 2013). Transient anterograde amnesia is a symptom occasionally observed in migraine. The inconsistent outcome between our study and others is probably related to the different time points when BDNF and TrkB expression were detected, or to the different inducers that evoke migraine and transient anterograde amnesia, respectively. BDNF is expressed in trigeminal ganglion neurons, which is in accordance with our IHC results, and it is released into the cerebrospinal fluid and blood after stimulation by trigeminal stimulation and nociceptive inputs. We observed that BDNF in the serum of NTG-induced migraine models was dramatically increased, both in the migraine attacks and headache-free intervals, compared to non-migraine control; and BDNF serum level was much higher in migraine attacks than in the headache-free intervals. Consistent to our data, clinical studies exhibited that migraine patients have significantly higher BDNF serum level during migraine attacks, compared with headache-free intervals and healthy controls (Fischer et al., 2012; Tanure et al., 2010). We also found that BDNF serum level was higher in the female than male rats in migraine attacks, implying a more profound role in female rats.

Ongoing studies have implicated neurogenic inflammation in pathogenesis of migraine. Peripheral and cerebrospinal fluid levels of pro-inflammatory cytokines have been found to be elevated in migraineurs. Intracranial administration of inflammatory mediators is also a common approach to induce migraine attacks, in addition to the peripheral administration of NTG (Sufka et al., 2016). In fact, NTG infusion in rats produced a delayed meningeal inflammation, as showed by the up-regulation of interleukin (IL)-1β, IL-6, TNF-α and inducible NO synthase (iNOS) (Reuter et al., 2001). BDNF is up-regulated in primary sensory neurons by inflammation. A study uncovered that the BDNF in the culture medium of trigeminal ganglion neuron is elevated following the cell exposure to TNF-α (Bałkowiec-Iskra et al., 2011). Besides, calcitonin gene-related peptide (CGRP) expression in trigeminal ganglia is increased with peripheral inflammatory reaction in the area of trigeminal nociceptors (Buldyrev et al., 2006). CGRP has recently been identified as a key player in the pathogenesis of migraine headaches. Importantly, CGRP enhances BDNF release from cultured trigeminal neurons in a dose-dependent manner, while the promoting effect is abolished when pretreated with CGRP receptor antagonist, CGRP (8-37) (Buldyrev et al., 2006). Thus, it is proposed that NTG-induced neurogenic inflammation acts as an important cause for BDNF up-regulation in migraine attacks.

Higher BDNF level has been correlated to increased glutamate levels in the cerebrospinal fluid of chronic migraine patients (Sarchielli et al., 2007). BDNF potentiates synaptic transmission in hippocampal area CA1, area CA3, and the dentate gyrus. In area CA3, BDNF potentiates a major glutamatergic input to pyramidal cells, the mossy fiber pathway (Scharfman and MacLusky, 2008). Glutamate acting as an enhanced excitatory neurotransmitter facilitates central sensitization (Gao et al., 2014). Trigeminal activation, resulting from both peripheral and central sensitization, is thought to be responsible for the headache phase of the migraine (Kuzawińska et al., 2014). Thus, the increased BDNF level might mediate NTG-triggered migraines probably through inducing trigeminal sensitization and activation. It is supported by previous research that observed a declined threshold for activation of neurons by stimulation of dural afferents after NTG infusion, and a facilitation of pain processing at the trigeminal level during NTG-triggered migraine attacks (Galeotti and Ghelardini, 2013).

Similar to BDNF, CREB is involved in sensitization of nociceptive cells and appears to be responsible for meningeal pain hypersensitivity (Galeotti and Ghelardini, 2013). There is evidence indicating that central sensitization within dorsal horn neurons is dependent on p-CREB transcriptional regulation (Mitsikostas et al., 2011). Inhibiting the nuclear translocation of CREB prevents the slowly developing onset of sensitization within the brainstem (Isensee et al., 2014). Activation of CREB within the trigeminal nucleus caudalis, or trigeminal ganglion *in vitro*, has been seen only after specific activation of nociceptive neurons, which implies that activation of CREB is implicated in pain transmission (Isensee et al., 2014). In the present study, p-CREB expression was found to be elevated during migraine attacks, but it was decreased in the following headache-free intervals. Our study thus suggested a positive correlation of p-CREB to migraine. p-CREB has been found to evoke neuronal presynaptic activation within the trigeminovascular system in animal models of migraine (Isensee et al., 2014). A study using IHC showed distribution of p-CREB within the pain-producing areas of the trigeminal system after capsaicin stimulation on meningeal artery; however, the p-CREB staining was attenuated when pre-treated with specific anti-migraine agents, like sumatriptan and naratriptan (Mitsikostas et al., 2011). Triptans, another family of agents against migraine, diminish the activity of CREB within the central parts of the trigeminal system, resulting in the inhibition of central sensitization and depression of brainstem nociceptive neurons, as has been determined using electrophysiological methods (Mitsikostas et al., 2011).

As an upstream modulator of CREB, ERK is thought to play a pivotal role in migraine pathology as phosphorylated ERK is involved in pain and nociceptive pathways and mediates neurogenic inflammation, stress and central sensitization. Documents noted that activation of the ERK pathway leads to the onset and maintenance of various pain conditions via transcriptional, translational or post-translational regulation (Zhang et al., 2013). IL-6 is a cytokine with an established role in modulating migraine. An increased phosphorylation of ERK in trigeminal ganglion neurons was observed with IL-6 application (Yan et al., 2012). Stress is one of the most consistently reported factors contributing to migraine, although the underlying mechanisms are not fully known. Stress caused by dural injection of norepinephrine, which is the primary sympathetic efferent transmitter involved in the headache phase of migraine, increased phosphorylation of ERK in rat trigeminal ganglia and dural fibroblasts (Wei et al., 2015). NTG injection evokes delayed meningeal nociceptor sensitization, which is accompanied with a robust ERK phosphorylation in meningeal arteries, but pharmacological blockade of meningeal ERK phosphorylation represses the development of NTG-evoked delayed meningeal nociceptor sensitization (Zhang et al., 2013). This study observed that NTG-induced migraine attacks elevated p-ERK level similar to its downstream effector, CREB. Thus, it is possible that ERK/CREB serves as an important signal in migraine.

**Fig. 8.**
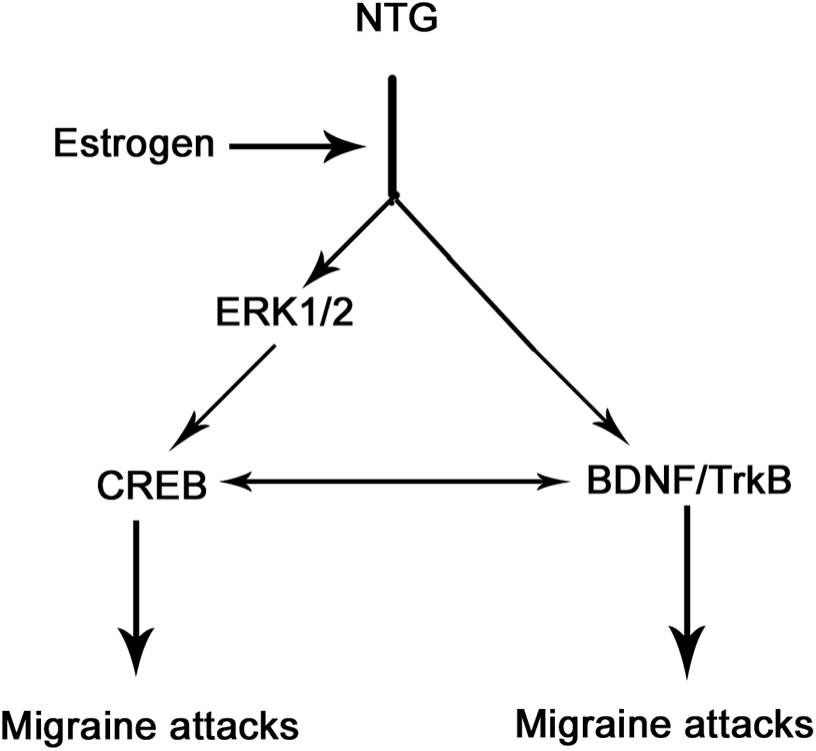
**Involvement of BDNF/TrkB and ERK/CREB axes in nitroglycerin-induced rat migraine and effects of estrogen on these signals in the migraine.** Nitroglycerin (NTC)-induced migraine attacks was accompanied by up-regulation of BDNF, TrkB, ERK and CREB in their expression and with enhanced the phosphorylation of ERK and CREB. These processes are promoted by estrogen, because the effect of NTC was attenuated in rats with ovariectomy, but not in ovariectomized rats that underwent exogenous estrogen administration. Expression or phosphorylation of BDNF, TrkB, ERK and CREB had remarkable reduction in the headache-free intervals compared to migraine attacks, suggesting that BDNF/TrkB and ERK/CREB axes participated in migraine attacks. BDNF, brain-derived neurotrophic factor; TrkB, tropomyosin receptor kinases; ERK, extracellular signal-regulated kinase; CREB, c-AMP-responsive element binding protein.

Estrogen is known to be a powerful trigger for migraine, but the endogenous factors that mediate the actions of estrogen in migraine are not fully understood. Fluctuations in serum estrogen levels are potentially relevant to migraine, because the symptoms often vary with the time of the ovarian cycle and the times of life when estradiol levels change dramatically, such as puberty, postpartum, or menopause (Scharfman and MacLusky, 2008). Migraine-related trigeminal pain is concentrated during the reproductive years, often beginning at menarche and declining after menopause (Scharfman and MacLusky, 2008). BDNF expression is elevated in the hippocampus as estradiol levels increased during the rodent estrous cycle, suggesting that BDNF is under the regulation of estrogen (Scharfman and MacLusky, 2008). The present study revealed the promoting effect of estrogen on the BDNF expression in NTG-induced migraine, based on the observation that estrogen depletion by ovariectomy resulted in BDNF reduction in NTG-induced migraine, whereas restoration of serum estrogen level via estrogen administration reversed the BDNF expression. Similar phenomena were also observed in TrkB, p-ERK, and p-CREB. Immunohistochemical studies have demonstrated the presence of estrogen receptor alpha (ERα) in nuclei of larger neurons and cytoplasm of smaller neurons, and the existence of the novel estrogen receptor G-protein coupled receptor 30 (GPR30) in small diameter neurons (Liverman et al., 2009). A study *in vitro* showed that specific agonists for ERα and GPR30 caused the activation of ERK in trigeminal ganglion neurons (Liverman et al., 2009). Given the positive correlation of BDNF, TrkB, p-ERK and p-CREB with NTG-induced migraine, which was confirmed by this and other studies (Fischer et al., 2012; Isensee et al., 2014; Zhang et al., 2013), these factors are probably involved in the actions of estrogen contributing to migraine, as described in the Fig. 8. BDNF/TrkB and ERK/CREB axes facilitate migraine attacks probably via sensitization of pain-sensing neurons (e.g. nociceptive neurons) in trigeminal nucleus caudalis and somatosensory cortex. Estrogen confers promoting effects on these axes, thus a high level of estrogen in serum is probably associated with an increased risk of migraine. Estrogen in serum is generally higher in the female than male, but it has notable variation in the menstrual cycle and reproductive period of female, which adds to the uncertainty when migraine attacks.

Collectively, this study unveiled a positive correlation of BDNF/TrkB and CREB/ERK axes in NTG-induced migraine and the promoting effects of estrogen on their signals in the migraine. Results obtained in this study contribute to understanding the pathogenesis of migraine and mechanisms by which estrogen contributes to migraine.

## MATERIALS AND METHODS

### Animals and ethics

A total of 84 Sprague-Dawley rats (24 males and 60 females, 6-8 weeks old, 180-220 g) were provided by the experimental animal center, Third Xiangya Hospital of Central South University (Changsha, China). The rats were housed four per cage (545 mm×359 mm×200 mm) and kept at 20–22°C on a 12 h light:12 h dark cycle with food and water *ad libitum*. This trial was approved by the local Animal Care Committee, and the animal welfare and experimental procedures were carried out in accordance with international ethical guidelines. In addition, the principles of the Helsinki declaration and International Association for the Study of Pain (IASP) guidelines for pain research in animals were rigorously applied.

### Experimental protocols

NTG was prepared by dissolving the stock solution of 5.0 mg/1.5 ml (Sigma, St Louis, MO, USA) in 27% alcohol and 73% propylene glycol (PG) and further diluting the dissolved solution in saline to reach the final concentration of 6% alcohol and 16% PG. The vehicle control used in these experiments was 16% PG, 6% alcohol and 0.9% saline. In experiment one, 24 male and 24 female Sprague–Dawley rats received intraperitoneal (i.p.) injection of the 10 mg/kg NTG or its vehicle once a week. The rats were sacrificed during the migraine attacks (4 h after the fifth injection) or the headache-free intervals (24 h after the fifth injection). For experiment two, 24 female Sprague–Dawley rats with ovariectomy underwent intraperitoneal administration of estrogen or its vehicle. Twelve rats of the sham-operated group only underwent the resection of a bit of fat around bilateral ovaries. All these rats were injected with 10 mg/kg NTG once a week and the injection was repeated five times.

### Behavioral evaluation

Migraine is characterized by recurrent headache attacks with a series of complex symptoms, like nausea, vomiting and sensitivity to light, sound or smell. As described by Sufka et al. (2016), rats presenting recurrent pathological manifestations, including painful facial action, head scratching, red ear, tail flick, and photophobia, after repeated NTG injections indicates that NTG successfully induces occurrence of migraine. Using a similar method, facial and behavioral characters of rats after the fifth injection with NTG were recorded to determine the effect of NTG on induction of migraine in the present study.

### Immunohistochemistry (IHC)

After undergoing behavioral testing, 5–8 rats per group were selected by randomization and assigned to subsequent IHC detection. Rats were anaesthetized with ketamine and xylazine (i.p.), and then the whole brain was removed and fixed in 5% paraformaldehyde for 3 h. The transverse sections of trigeminal ganglion samples were incubated in 0.3% Triton X-100 and 2% normal goat serum for 1 h, followed by incubation with antibodies against BDNF, TrkB, p-CREB, and p-Erk (Santa Cruz Biotechnology). Following incubation, sections were thoroughly washed with PBS and incubated with biotinylated secondary antibody and avidin-biotin complex (Vectastain – ABC kit PK-6100 Elite, 1 h) for color detection.

### Real-time polymerase chain reaction (RT-PCR)

After rats were killed, their trigeminal ganglion were isolated from brains and immediately frozen at −80°C until further processing. Total RNA was isolated from the cerebral samples with Trizol reagent (Invitrogen), and then cDNA was generated using the iScript cDNA Synthesis kit (Bio-Rad) following the supplier's instructions. Details of primers used for quantitative RT-PCR was presented in Table 4. The amplification was performed through two-step cycling (95–60°C) for 45 cycles in a light Cycler 480 Instrument RT-PCR Detection System (Roche) following the supplier's instructions. All samples were assayed in triplicate. Gene expression was calculated using the −ΔCt method.

**Table 4.**
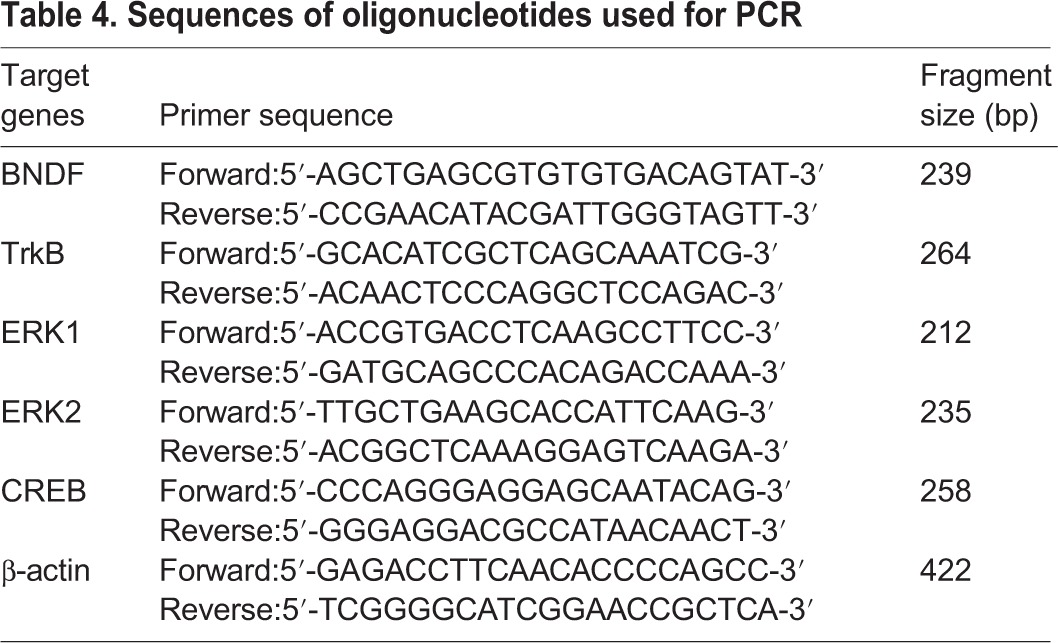
**Sequences of oligonucleotides used for PCR**

### Western blot assay

The trigeminal ganglion isolated from brain were homogenized on ice within a modified RIPA buffer (Tris 50 mM, pH 7.4, NaCl 150 mM, EDTA 1 mM, SDS 0.2%) supplemented with cocktail inhibitors protease. 20 μg of protein was submitted to 10% SDS-poliacrylamide gels and transferred onto a PVDF membrane (Amersham Biosciences). After blocking with 5% dry milk, the membrane was incubated overnight at 4°C with primary antibodies against BDNF, TrkB, p-CREB, and p-Erk (Santa Cruz Biotechnology). Blots in the membrane were probed with a horseradish peroxidase coupled secondary antibody (1:10,000; Amersham Biosciences). An enhanced chemiluminescence system (ECL Advance; Amersham Biosciences) was used for visualization.

### Enzyme-linked immunosorbant assays (ELISA)

Rats' blood was drawn from the vena cava before the sacrifice, and then centrifuged at 3000 ***g*** for 10 min at 4°C to separate serum. In experiment one, the level of BDNF in serum was quantified by ELISA kit (Highly Sensitive R&D Systems, Minneapolis, MN, USA), according to the manufacturer's instructions. ELISA kit (USCN, Wuhan, China) was used to detect the serum estrogen level in experiment two. Precision of the assay was verified by determination of the inter- and intra-assay coefficients of variation.

### Statistical analyses

All the data were expressed as mean±s.d. Data analysis was tested by one-way analysis of variance (ANOVA) with IBM SPSS (version 19.0, SPSS Inc, Chicago, IL, USA). ANOVA followed by a Tukey's post hoc correlation for multiple comparisons. *P*<0.05 was established as significant difference.

## References

[BIO021022C1] Bałkowiec-Iskra E., Vermehren-Schmaedick A. and Balkowiec A. (2011). Tumor necrosis factor-α increases brain-derived neurotrophic factor expression in trigeminal ganglion neurons in an activity-dependent manner. *Neuroscience* 180, 322-333. 10.1016/j.neuroscience.2011.02.028 21335064 PMC3070813

[BIO021022C2] Bhatt D. K., Ramachandran R., Christensen S. L., Gupta S., Jansen-Olesen I. and Olesen J. (2015). CGRP infusion in unanesthetized rats increases expression of c-Fos in the nucleus tractus solitarius and caudal ventrolateral medulla, but not in the trigeminal nucleus caudalis. *Cephalalgia* 35, 220-233. 10.1177/0333102414535995 24895375

[BIO021022C3] Borkum J. M. (2016). Migraine triggers and oxidative stress: a narrative review and synthesis. *Headache* 56, 12-35. 10.1111/head.12725 26639834

[BIO021022C4] Buldyrev I., Tanner N. M., Hsieh H.-Y., Dodd E. G., Nguyen L. T. and Balkowiec A. (2006). Calcitonin gene-related peptide enhances release of native brain-derived neurotrophic factor from trigeminal ganglion neurons. *J. Neurochem.* 99, 1338-1350. 10.1111/j.1471-4159.2006.04161.x 17064360 PMC2440676

[BIO021022C5] Cho H.-J., Sung Y.-H., Lee S.-H., Chung J.-Y., Kang J.-M. and Yi J.-W. (2013). Isoflurane induces transient anterograde amnesia through suppression of brain-derived neurotrophic factor in hippocampus. *J. Korean Neurosurg. Soc.* 53, 139-144. 10.3340/jkns.2013.53.3.139 23634262 PMC3638265

[BIO021022C6] Fischer M., Wille G., Klien S., Shanib H., Holle D., Gaul C. and Broessner G. (2012). Brain-derived neurotrophic factor in primary headaches. *J. Headache Pain* 13, 469-475. 10.1007/s10194-012-0454-5 22584531 PMC3464472

[BIO021022C7] Galeotti N. and Ghelardini C. (2013). Inhibition of the PKCγ-ε pathway relieves from meningeal nociception in an animal model: an innovative perspective for migraine therapy? *Neurotherapeutics* 10, 329-339. 10.1007/s13311-012-0151-8 23055050 PMC3625380

[BIO021022C8] Gao Z., Liu X., Yu S., Zhang Q., Chen Q., Wu Q., Liu J., Sun B., Fang L., Lin J. et al. (2014). Electroacupuncture at acupoints reverses plasma glutamate, lipid, and LDL/VLDL in an acute migraine rat model: a (1) H NMR-based metabolomic study. *Evid. Based Complement Alternat. Med.* 2014, 659268 10.1155/2014/659268 24592282 PMC3921982

[BIO021022C9] Gölöncsér F. and Sperlágh B. (2014). Effect of genetic deletion and pharmacological antagonism of P2X7 receptors in a mouse animal model of migraine. *J. Headache Pain* 15, 24 10.1186/1129-2377-15-24 24885962 PMC4016653

[BIO021022C10] Greco R., Tassorelli C., Mangione A. S., Smeraldi A., Allena M., Sandrini G., Nappi G. and Nappi R. E. (2013). Effect of sex and estrogens on neuronal activation in an animal model of migraine. *Headache* 53, 288-296. 10.1111/j.1526-4610.2012.02249.x 22913654

[BIO021022C11] Greco R., Mangione A. S., Sandrini G., Nappi G. and Tassorelli C. (2014). Activation of CB2 receptors as a potential therapeutic target for migraine: evaluation in an animal model. *J. Headache Pain* 15, 14 10.1186/1129-2377-15-14 24636539 PMC3995520

[BIO021022C12] Hong E. J., McCord A. E. and Greenberg M. E. (2008). A biological function for the neuronal activity-dependent component of Bdnf transcription in the development of cortical inhibition. *Neuron* 60, 610-624. 10.1016/j.neuron.2008.09.024 19038219 PMC2873221

[BIO021022C13] Isensee J., Diskar M., Waldherr S., Buschow R., Hasenauer J., Prinz A., Allgöwer F., Herberg F. W. and Hucho T. (2014). Pain modulators regulate the dynamics of PKA-RII phosphorylation in subgroups of sensory neurons. *J. Cell Sci.* 127, 216-229. 10.1242/jcs.136580 24190886

[BIO021022C14] Kuzawińska O., Lis K., Cudna A. and Bałkowiec-Iskra E. (2014). Gender differences in the neurochemical response of trigeminal ganglion neurons to peripheral inflammation in mice. *Acta Neurobiol. Exp.* 74, 227-232. 10.55782/ane-2014-1988 24993632

[BIO021022C15] Liverman C. S., Brown J. W., Sandhir R., McCarson K. E. and Berman N. E. J. (2009). Role of the oestrogen receptors GPR30 and ERalpha in peripheral sensitization: relevance to trigeminal pain disorders in women. *Cephalalgia* 29, 729-741. 10.1111/j.1468-2982.2008.01789.x 19220308 PMC4054707

[BIO021022C16] Merki-Feld G. S., Imthurn B., Langner R., Seifert B. and Gantenbein A. R. (2015). Positive effects of the progestin desogestrel 75 μg on migraine frequency and use of acute medication are sustained over a treatment period of 180 days. *J. Headache Pain* 16, 39 10.1186/s10194-015-0522-8 PMC4420760 25933634

[BIO021022C17] Mitsikostas D. D., Knight Y. E., Lasalandra M., Kavantzas N. and Goadsby P. J. (2011). Triptans attenuate capsaicin-induced CREB phosphorylation within the trigeminal nucleus caudalis: a mechanism to prevent central sensitization? *J. Headache Pain* 12, 411-417. 10.1007/s10194-011-0352-2 21626018 PMC3139063

[BIO021022C18] Reuter U., Bolay H., Jansen-Olesen I., Chiarugi A., Sanchez del Rio M., Letourneau R., Theoharides T. C., Waeber C. and Moskowitz M. A. (2001). Delayed inflammation in rat meninges: implications for migraine pathophysiology. *Brain* 124, 2490-2502. 10.1093/brain/124.12.2490 11701602

[BIO021022C19] Sarchielli P., Mancini M. L., Floridi A., Coppola F., Rossi C., Nardi K., Acciarresi M., Pini L. A. and Calabresi P. (2007). Increased levels of neurotrophins are not specific for chronic migraine: evidence from primary fibromyalgia syndrome. *J. Pain* 8, 737-745. 10.1016/j.jpain.2007.05.002 17611164

[BIO021022C20] Scharfman H. E. and MacLusky N. J. (2008). Estrogen-growth factor interactions and their contributions to neurological disorders. *Headache* 48, S77-S89. 10.1111/j.1526-4610.2008.01200.x 18700946 PMC2729400

[BIO021022C21] Simonetti M., Giniatullin R. and Fabbretti E. (2008). Mechanisms mediating the enhanced gene transcription of P2X3 receptor by calcitonin gene-related peptide in trigeminal sensory neurons. *J. Biol. Chem.* 283, 18743-18752. 10.1074/jbc.M800296200 18460469

[BIO021022C22] Sufka K. J., Staszko S. M., Johnson A. P., Davis M. E., Davis R. E. and Smitherman T. A. (2016). Clinically relevant behavioral endpoints in a recurrent nitroglycerin migraine model in rats. *J. Headache Pain.* 17, 40 10.1186/s10194-016-0624-y 27093871 PMC4837195

[BIO021022C23] Tanure M. T. A., Gomez R. S., Hurtado R. C. L., Teixeira A. L. and Domingues R. B. (2010). Increased serum levels of brain-derived neurotropic factor during migraine attacks: a pilot study. *J. Headache Pain.* 11, 427-430. 10.1007/s10194-010-0233-0 20556464 PMC3452273

[BIO021022C24] Tong L., Thornton P. L., Balazs R. and Cotman C. W. (2001). Beta-amyloid-(1–42) impairs activity-dependent cAMP-response element-binding protein signaling in neurons at concentrations in which cell survival is not compromised. *J. Biol. Chem.* 276, 17301-17306. 10.1074/jbc.M010450200 11278679

[BIO021022C25] Wei X., Yan J., Tillu D., Asiedu M., Weinstein N., Melemedjian O., Price T. and Dussor G. (2015). Meningeal norepinephrine produces headache behaviors in rats via actions both on dural afferents and fibroblasts. *Cephalalgia* 35, 1054-1064. 10.1177/0333102414566861 25601915 PMC4506895

[BIO021022C26] Yan J., Melemedjian O. K., Price T. J. and Dussor G. (2012). Sensitization of dural afferents underlies migraine-related behavior following meningeal application of interleukin-6 (IL-6). *Mol. Pain* 8, 6 10.1186/1744-8069-8-6 22273495 PMC3274468

[BIO021022C27] Yang Y., Ligthart L., Terwindt G. M., Boomsma D. I., Rodriguez-Acevedo A. J. and Nyholt D. R. (2016). Genetic epidemiology of migraine and depression. *Cephalalgia* 36, 679-691. 10.1177/0333102416638520 26966318

[BIO021022C28] Zha X.-M., Bishop J. F., Hansen M. R., Victoria L., Abbas P. J., Mouradian M. M. and Green S. H. (2001). BDNF synthesis in spiral ganglion neurons is constitutive and CREB-dependent. *Hear. Res.* 156, 53-68. 10.1016/S0378-5955(01)00267-2 11377882

[BIO021022C29] Zhang X., Kainz V., Zhao J., Strassman A. M. and Levy D. (2013). Vascular extracellular signal-regulated kinase mediates migraine-related sensitization of meningeal nociceptors. *Ann. Neurol.* 73, 741-750. 10.1002/ana.23873 23447360 PMC3688635

